# A percolation theory analysis of continuous functional paths in protein sequence space affirms previous insights on the optimization of proteins for adaptability

**DOI:** 10.1371/journal.pone.0314929

**Published:** 2024-12-05

**Authors:** Brian J. Miller

**Affiliations:** Biologic Institute, Redmond, WA, United States of America; University of Vermont College of Medicine, UNITED STATES OF AMERICA

## Abstract

A key question in protein evolution and protein engineering is the prevalence of evolutionary paths between distinct proteins. An evolutionary path corresponds to a continuous path of functional sequences in sequence space leading from one protein to another. Natural selection could direct a mutating coding region in DNA along a continuous functional path (CFP), so a new protein could arise far more easily than if a coding region were randomly mutating without any constraints. The distribution and length of CFPs undergird theories on the origin of natural proteins and strategies for engineering artificial proteins. This study examined the distribution of long CFPs within the framework of percolation theory, which addresses the proportion of randomly filled sites in a lattice above which long continuous paths of neighboring filled sites become common (aka percolation threshold). It also used a simulation to demonstrate that the percolation threshold in protein sequence space approximates the reciprocal of the average number of protein variants that could result from a single mutation. For diverse proteins, the ratio was calculated between the percolation threshold and the proportion of sequences reported to perform a protein’s function, relative to the total number of sequences of that protein’s length. This ratio represents a measure of the biasing in the distribution of functional sequences required for evolutionary paths to possibly exist, so it provides a means to quantify the specificity in protein sequence and structure required to allow for a protein to develop new catalytic functions. The consistently high ratio demonstrates that CFPs can only connect distinct proteins if the biasing in the distribution of functional sequences in sequence space is often extremely large. Regions in sequence space are identified where the biasing is sufficient to allow for extensive CFPs. The calculated levels of required biasing and the identified regions of high biasing reinforce the conclusion of previous studies that some proteins are highly optimized, so mutations can enable or enhance catalytic functions while maintaining the protein’s structure. The conclusions of this study also challenge the results of a previous application of percolation theory to sequence space that did not properly incorporate the percolation threshold. Steps are outlined for integrating the percolation threshold and the biasing measure into studies of protein sequence space.

## Introduction

### Background

A central question in protein engineering and protein evolution is how new proteins can emerge de novo or through the modification of existing proteins. The answer depends largely on two key factors. The first is the prevalence of amino acid sequences that perform a particular function. The prevalence can be determined for all of sequence space, which is the multidimensional space of all possible amino acid sequences. The proportion of functional sequences, *P*_*fs*_, is then defined as the number of sequences that perform the protein’s function and maintain its structure divided by the total number of sequences with the protein’s length. The proportion of functional sequences can also be defined in a local region of sequence space. The local proportion, *P*_*loc*_, is the number of functional sequences divided by the total number of sequences in that region.

The variables used in this study are listed in Table 1 in their order of appearance.

**Table 1:** List of variables.

Variable	Description
P_fs_	Proportion of functional sequences: Number of functional sequences divided by the total number of sequences in sequence space.
P_loc_	Local proportion of functional sequences: Number of functional sequences divided by the total number of sequences in a local region of sequence space.
n_m_	Number of mutations: Maximum number of mutations separating two sequences that are considered neighbors in a continuous functional path (CFP) or in a cluster.
P_th_	Percolation Threshold: The proportion of functional sequences below which CFPs that extend long distances in sequence space cease to exist.
z	Number of neighbors: Number of sites neighboring a site in a lattice or average number of sequences neighboring a sequence in sequence space.
L	Length: Number of amino acids in a peptide, polypeptide, or protein.
A_t_	Amino acid transitions: Average number of amino acids into which an amino acid could transition from a single mutation.
R_b_	Biasing Ratio: Ratio of the percolation threshold to the proportion of functional sequences.
σσ	Standard Deviation: The standard deviation of the normal distribution used to bias the proportion of functional sequences in Fig 2.
A	Number of amino acids: Number of amino acids that could reside at each location in a sequence in the simulation of sequence space.
N_att_	Average number of attempts: Average number of trial matrices that were required to generate one with a CFP connecting a starting sequence to a target.
n	Number of differences: Number of amino acid differences between a given sequence and a wildtype sequence.
n_max_	Maximum number of differences: Maximum number of amino acid differences between a sequence and a wildtype sequence where *P*_*loc*_*(n)* still exceeds *P*_*th*_.
P_tol_	Percentage of tolerated mutations: The percentage of mutations that do not disable a β-lactamase enzyme from Bershtein et al. (2006).
N(s)	Number of sequences in cluster: The number of sequences in clusters of size *s* as a function of *s* from Buchholz et al. (2017).

A second key factor is the distribution in sequence space of paths of functional neighboring sequences, which are termed continuous functional paths (CFPs). A neighboring sequence typically differs from an initial sequence by a single mutation. A continuous path is the series of neighboring sequences that results from a series of specific mutations. A CFP is a continuous path where every sequence in the path is functional.

In certain contexts, neighbors are defined as those sequences that are separated by not just one but up to a set number of mutations, *n*_*m*_. A CFP is then defined as a series of functional sequences that results from a series of sets of *n*_*m*_ or fewer mutations. For instance, in species with very large populations, individuals can acquire multiple mutations at once, so *n*_*m*_ would represent the maximum possible number of simultaneous mutations. Relatedly, nonessential proteins might acquire a disabling mutation but persist in the species for many generations. The protein could potentially acquire an additional mutation or mutations that reactivated it before natural selection removed the initial mutation from the population. In this situation, *n*_*m*_ would represent the maximum number of mutations that a protein could potentially acquire within the available timeframe to regain function if the protein lost function with the first *n*_*m*_− 1 mutations. As a final example, an insertion/deletion (indel) could add or remove multiple amino acids in a single event, so *n*_*m*_ would represent the number of amino acids that an indel adds or removes. Indels that do more than add or remove a few amino acids are almost always harmful [1], so they can be ignored.

**Fig 1:**
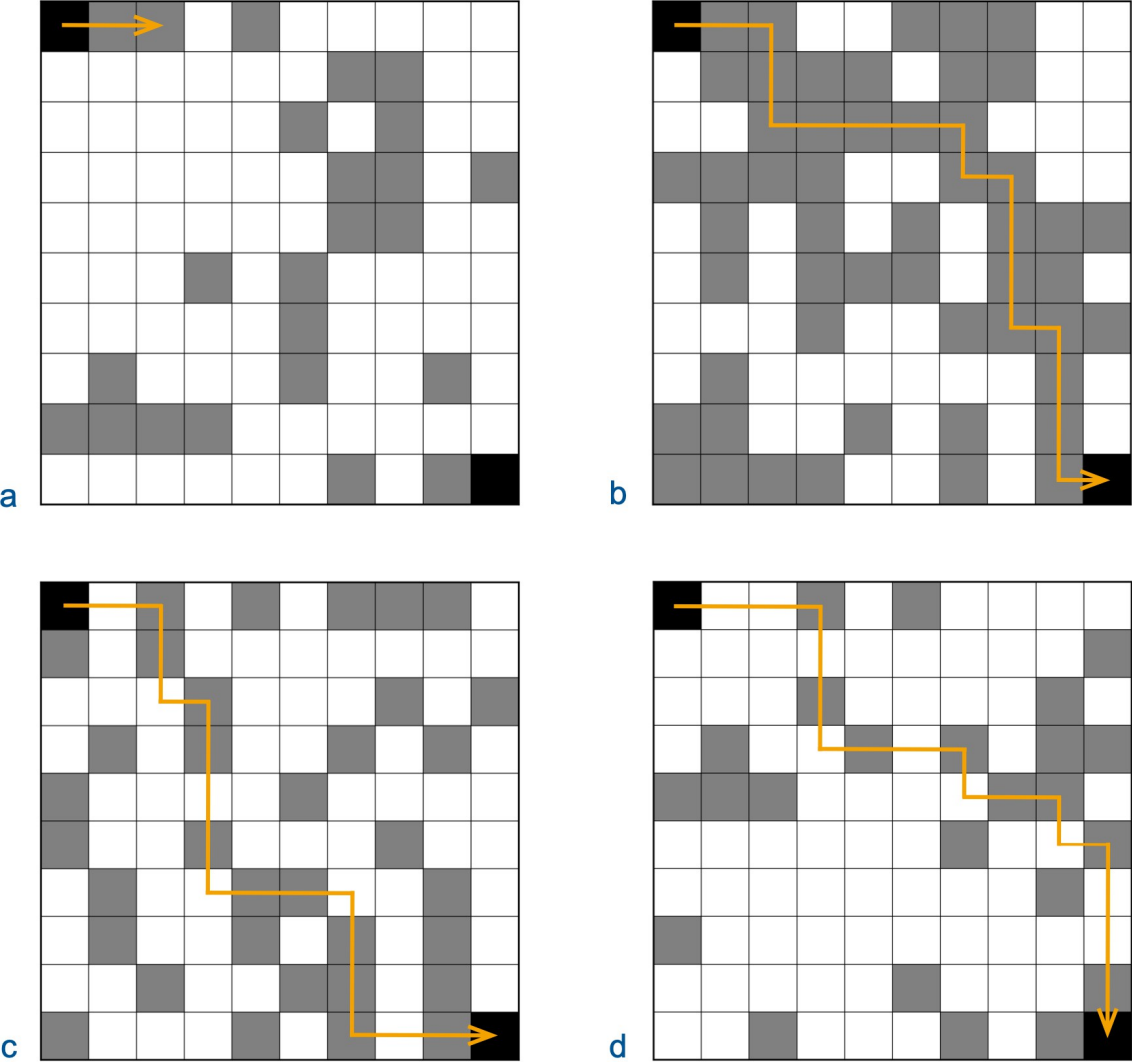
Continuous functional paths. For illustrative purposes, a small region of protein sequence space is depicted as a 10 x 10 grid where sequences that differ by a single mutation are directly above, below, to the left, or to the right of each other. Each sequence has a probability *P*_*loc*_ of being functional. Functional sequences are depicted as grey squares, and a starting sequence (top-left) and an ending sequence (bottom-right) are depicted as black squares. Neighboring sequences are within a certain number of mutations, *n*_*m*_, of each other. Continuous paths of functional sequences (CFPs) are identified by orange turning arrows. The *P*_*loc*_ and the *n*_*m*_ for the identified CFP are listed for each grid. (a) *P*_*loc*_ = 30%, *n*_*m*_ = 1. Only one CFP extends from the starting sequence, and no CFPs extend for significant distances. (b) *P*_*loc*_ = 50%, *n*_*m*_ = 1. Multiple CFPs connect the starting and ending sequences. As *P*_*loc*_ increases, the number and average length of CFPs also increase. (c) *P*_*loc*_ = 30%, *n*_*m*_ = 2. No CFPs of immediate neighbors (*n*_*m*_ = 1) connect the starting and ending sequences. CFPs do connect the starting and ending sequence if one nonfunctional sequence can reside between two functional sequences (*n*_*m*_ = 2). (d) *P*_*loc*_ = 30%, *n*_*m*_ = 3. No CFPs connect the starting and ending sequence for *n*_*m*_ = 1 or 2, but CFPs connect them for *n*_*m*_ = 3.

Examples of CFPs for different *P*_*loc*_ and *n*_*m*_ are illustrated in Fig 1. The diagrams display how a protein’s *P*_*loc*_ correlates with the number and average length of CFPs and how lower *P*_*loc*_ require higher *n*_*m*_ for CFPs to extend significant distances. The value of *P*_*loc*_ in different regions of sequence space constrain theories of protein evolution and strategies for engineering new proteins.

Information about CFPs is critical to understanding how new proteins emerge since many proteins correspond to *P*_*fs*_ so small that they could not have arisen through an undirected search in sequence space. The *P*_*fs*_ values commonly cited for peptides, polypeptides, and proteins are listed in Table 2. By comparison, the largest number of possible variants of a protein in all organisms for the entire history of the earth is on the order of 10^38^ [2]. For a protein to have arisen through random mutations in a freely evolving coding region of DNA, its *P*_*fs*_ must be larger than the reciprocal of this value (10^−38^). Yet the reported *P*_*fs*_ for most proteins is close to or smaller than this cutoff.

**Table 2:** Comparison of *P*_*th*_ to *P*_*fs*_. The table lists for several peptides, polypeptides, and proteins their length (*L*), percolation threshold (*P*_*th*_), proportion of functional sequences (*P*_*fs*_), and the ratio of the percolation threshold to the proportion of functional sequences (*R*_*b*_). It also lists the minimum allowed number of mutations between neighboring sequences (*n*_*min*_) for *P*_*th*_ to approximate or drop below *P*_*fs*_. The *n*_*min*_ values were converted to sequence identities (SI) using Eq 4. The study that reported a *P*_*fs*_ value is cited next to the protein’s name. Proteins are listed in order of descending *P*_*fs*_.

Protein/Peptide	Length	P_th_	P_fs_	R_b_	n_min_	SI
Membrane Embedding [3]	50	.0027	10^−8^	10^5^	4	92%
ATP Binding [4]	80	.0017	10^−12^	10^9^	5	94%
Chorismate Mutase [5]	99	.0013	10^−23^	10^20^	11	89%
WW [6]	35	.0038	10^−24^	10^21^	15	57%
Villin [6]	35	.0038	10^−33^	10^30^	23	34%
Titan I27 [6]	89	.0015	10^−38^	10^35^	20	78%
TNfn3 [6]	90	.0015	10^−39^	10^36^	21	77%
PDZ [6]	94	.0014	10^−50^	10^47^	27	71%
NTL9 [6]	56	.0024	10^−54^	10^51^	37	34%
λ-repressor [7]	92	.0014	10^−63^	10^60^	29	68%
Cytochrome c [8]	100	.0013	10^−65^	10^62^	38	62%
β-lactamase Domain [9]	153	.00087	10^−77^	10^74^	42	73%
IM7 [6]	87	.0015	10^−86^	10^74^	59	32%
IFABP [6]	131	.0010	10^−111^	10^108^	71	46%
α-LA [6]	123	.0011	10^−121^	10^118^	83	33%
OmpA [6]	171	.00078	10^−126^	10^123^	77	55%

For *P*_*fs*_ below the cutoff, an evolutionary search could only discover a functional sequence if it explored a vastly smaller portion of sequence space than what would have been required for an unconstrained randomly mutating sequence. If a CFP connected two proteins, natural selection could constrain a search along the CFP making the evolution of one protein into the other feasible. The existence of CFPs that extend long distances in sequence space depends on whether *P*_*loc*_ is above what is termed the percolation threshold, *P*_*th*_. When *P*_*loc*_ rises above the threshold, a phase change occurs where long CFPs become common. Below the threshold, CFPs are almost always only a few sequences in length. The value of the threshold in different contexts has been a central subject of study in percolation theory.

Extensive research has been conducted on identifying *P*_*th*_ for multidimensional latices, and multiple studies demonstrated that it approximates the reciprocal of the number of nearest neighboring sites, *z*, to a site inside a lattice [10, 11]:

$$P_{th}\approx \frac{1}{z}.$$ (1)

For example, if sites inside a multidimensional lattice had *z* = 100 neighbors, the percolation threshold would approximate 1/100 or 1%. Gavrilets (2003) derived the same equation for genotype space, where *z* represents the number of genotypes accessible through a single mutation [12].

This relationship should also hold in amino acid sequence space. For sequences of length *L*, the average number of nearest neighbors (sequences accessible from a single mutation) to any sequence is *A*_*t*_*L*, where *A*_*t*_ is the average number of amino acids an amino acid could transition into through a single mutation. By extension, the number of neighbors within *n*_*m*_ mutations is *A*_*t*_*L* to the power of *n*_*m*_ divided by *n*_*m*_!. For multiple mutations, the number of neighbors (*z*) is raised to the power of *n*_*m*_ since each mutation could make any possible change and sequences that are reached by fewer than *n*_*m*_ mutations are also neighbors. The factorial term is required since the order of alterations does not matter. An approximation for *P*_*th*_ directly follows from Eq 1:

$$P_{th}\approx \frac{n_{m}!}{{\left(A_{t}N\right)}^{n_{m}}}.$$ (2)

**Fig 2:**
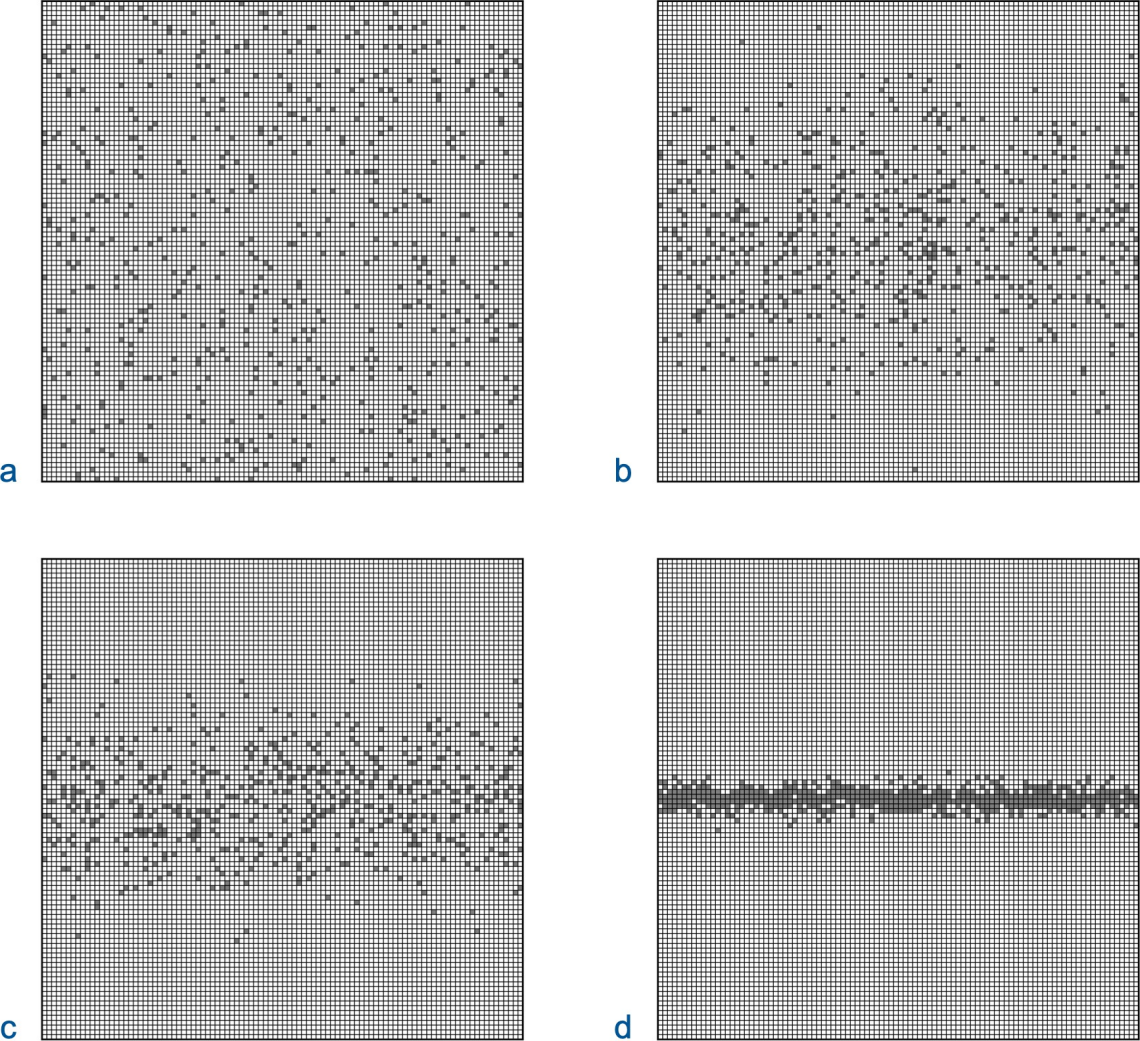
Biasing of functional sequences in sequence space. Sequence space is depicted as a 100 x 100 grid with neighboring sequences of a given sequence located directly above, below, to the left, and to the right. The proportion of functional sequences, *P*_*fs*_, in every grid is close to 5%. The functional sequences are depicted as grey squares. The local probability of a sequence being functional, *P*_*loc*_, is weighted along the y-axis by a normal distribution centered in the middle with a standard deviation of σ. The σ of the weighting function and the ratio, *R*_*b*_, of *P*_*loc*_ in the center to *P*_*fs*_ are listed for each grid. (a) σ = infinity, *R*_*b*_ = 1. Functional sequences are distributed uniformly throughout the grid. (b) σ = 15, *R*_*b*_ = 1.6. (c) σ = 10, *R*_*b*_ = 4.0. (d) σ = 2, *R*_*b*_ = 19. Only the last grid contains continuous functional paths extending from the grid’s left side to its right side.

If a protein’s *P*_*fs*_ is considerably below its *P*_*th*_, the distribution of functional sequences in local regions of sequence space must be highly biased for *P*_*loc*_ to be sufficiently large for CFPs to extend significant distances (Fig 2). A measure of the level of required biasing is the ratio, *R*_*b*_, of the percolation threshold to the proportion of functional sequences:

$$R_{b}=\frac{P_{th}}{P_{fs}}.$$ (3)

If *R*_*b*_ were 100 in a corridor extending through sequence space, *P*_*loc*_ would need to be at least 100 times larger than *P*_*fs*_ for a CFP to have a significant chance of extending through the corridor.

### Current study

The current study applies percolation theory to protein sequence space for diverse proteins to determine the level of required biasing in the distribution of functional sequences to allow for CFPs to extend for long distances. Quantifying the biasing requires identifying the percolation threshold in protein sequence space, so a simulation was developed to confirm that Eq 2 reliably approximates proteins’ *P*_*th*_. The simulation generated matrices that model protein sequence space for short amino acid chains, and it tested for the presence of CFPs for varying *P*_*fs*_. The simulation results demonstrate that Eq 2 accurately predicts the percolation threshold, so it was used to calculate *R*_*b*_ for several peptides, polypeptides, and proteins.

All proteins included in the analysis demonstrate very high *R*_*b*_ values, indicating that long CFPs can only exist in regions of sequence space with high levels of biasing. Sources of strong biasing were identified based on protein mutation studies and protein evolution research. The results of this study reinforce the conclusion of previous studies that many proteins possess highly special features that allow them to evolve new catalytic functions.

In addition, a new approach is proposed for incorporating *R*_*b*_ values into analyses of protein sequence space. Previous studies applying percolation theory to biological research have typically focused on genomic sequence space and mutations’ impact on physical traits [13–16] instead of individual proteins. Investigations that applied percolation theory to proteins typically studied their physical structure [17, 18]. The conceptual lattice corresponded to the possible positions of amino acids in a protein’s molecular architecture, and neighboring cells corresponded to amino acids that directly interact instead of amino acid sequences that are separated in sequence space by a set number of mutations.

One of the only studies that employed percolation theory to understand protein sequence space is Buchholz et al. (2017). The investigators concluded that a single cluster of functional sequences likely connects all proteins in the same superfamily [19], and a few sequences within these extensive clusters have many neighbors. These “hub sequences” are expected to tolerate multiple mutations that may give rise to new functions [20, 21]. The investigators suggest that these sequences could serve as promising starting points for directed evolution experiments [22]. Their methodology, however, did not account for the percolation threshold, an omission that raises questions about the validity of their conclusions.

No identified methodology properly applied percolation theory to protein sequence space to ascertain CFPs. The new approach fills this gap; it should lead to more accurate evaluations of the distributions of functional sequences, extensive CFPs, and large clusters. Steps are outlined for applying the approach to protein evolution and protein design studies.

## Results

### Simulation

**Fig 3:**
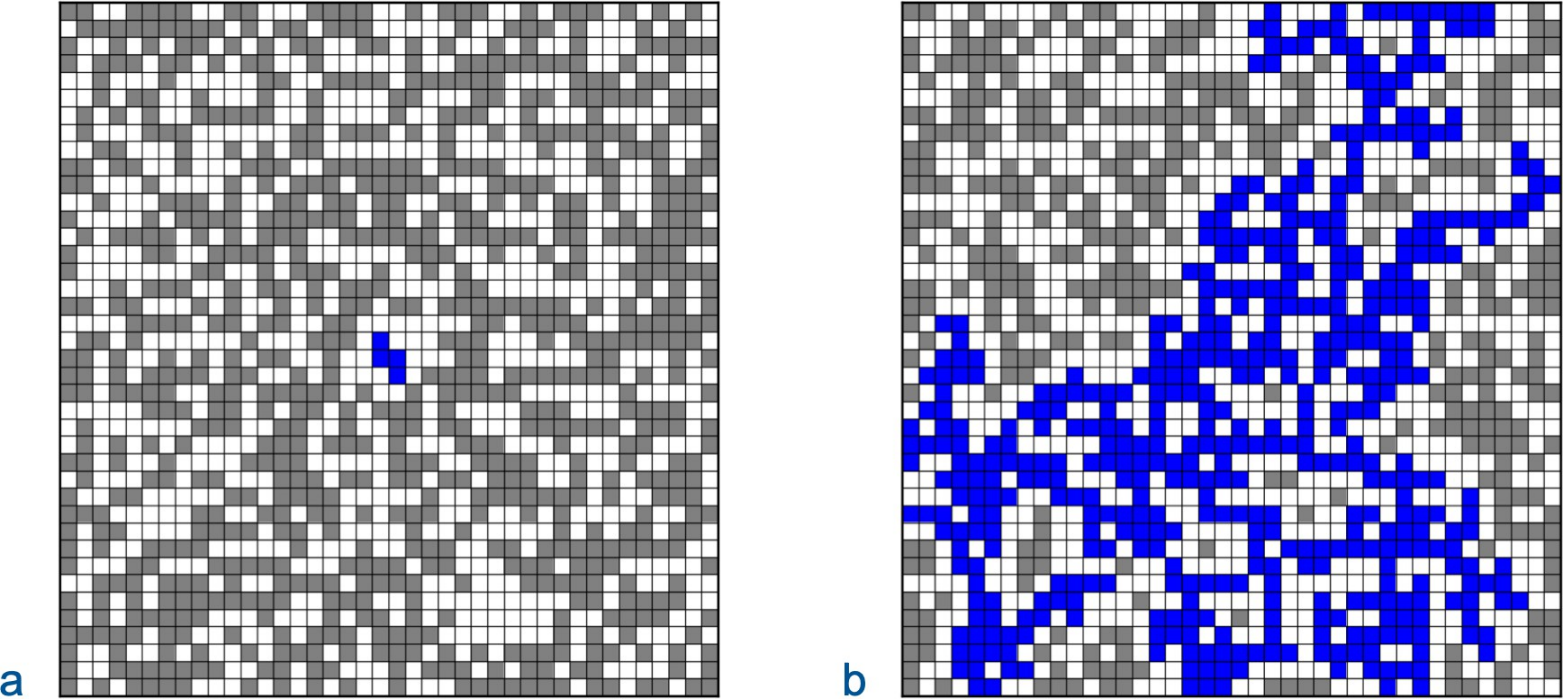
Clusters including center sequence. Sequence space is depicted as a 40 x 40 grid where 50% of the sequences were randomly assigned as functional. The functional sequences are depicted by grey squares. Neighboring sequences are above, below, to the left, and to the right. A cluster is the set of sequences that are connected to each other through continuous functional paths. Sequences in the center clusters that include the square (20, 20) are colored blue. (a) The center cluster only includes 4 sequences. (b) The center cluster extends throughout the grid.

The simulation generated multidimensional matrices corresponding to the sequence space for amino acid chains of length, *L*, where each position could correspond to any of *A* amino acids. In most trials, each amino acid could directly transition into any other amino acid, but not to itself, so the number of transition options, *A*_*t*_, was *A* – 1. For each trial, a matrix was generated with a set proportion of functional sequences, *P*_*fs*_, uniformly distributed. For one set of trials, the simulation calculated the size of the cluster of neighboring functional sequences that included the starting sequence. A cluster is all sequences that are connected to each other through CFPs (Fig 3). For another set of trials, the simulation determined if a CFP (*n*_*m*_ = 1) extended from a starting sequence to a target.

**Fig 4:**
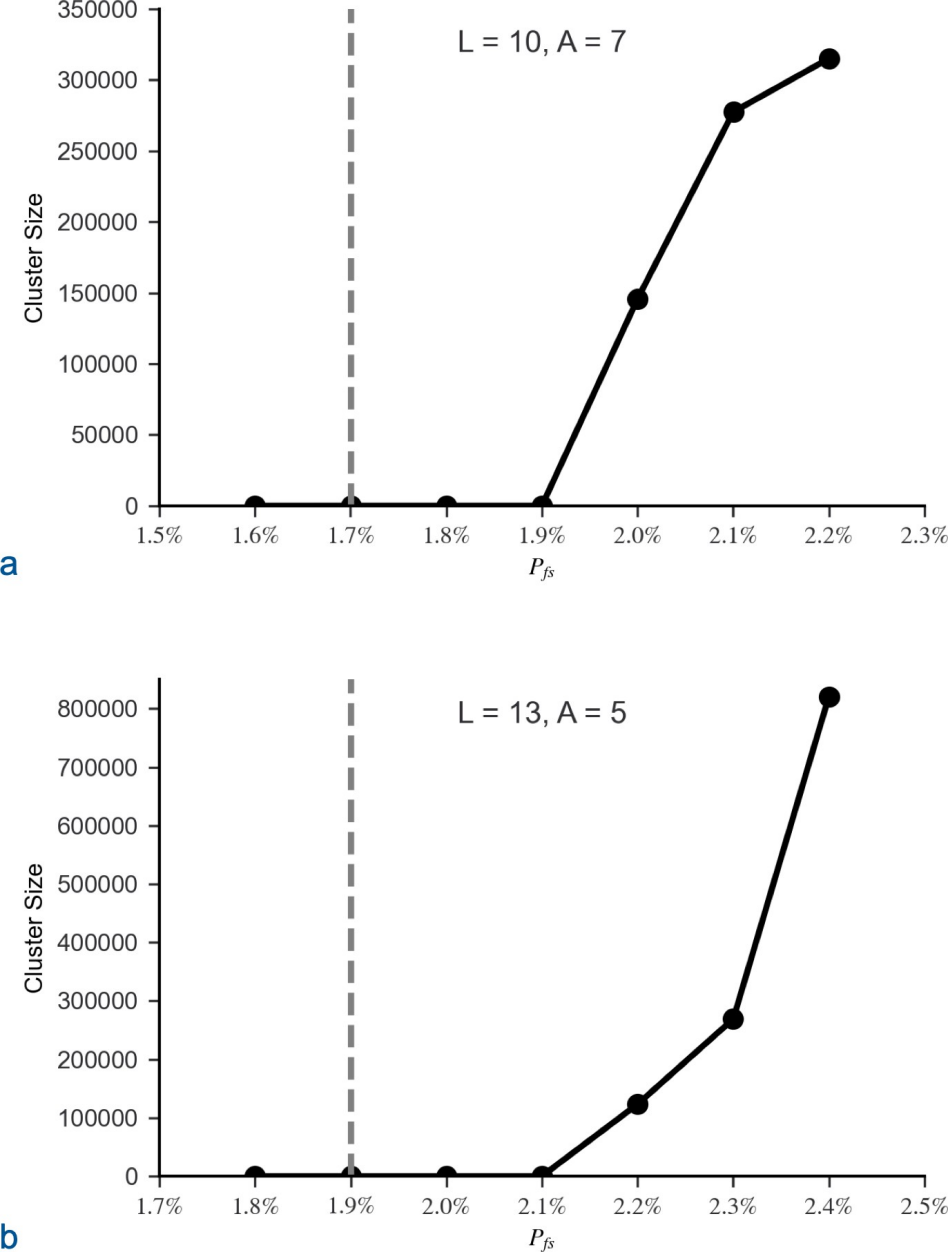
Average size of clusters of functional sequences that include the starting sequence. The average cluster size that included the starting sequence was calculated for 20 matrices randomly populated with the same proportion of functional sequences, *P*_*fs*_. The average cluster size increased dramatically after *P*_*fs*_ rose above a critical value, which is approximately 0.2% above the estimated percolation threshold, *P*_*th*_ (Eq 2: *n*_*m*_ = 1), for both (a) *L* = 10, *A* = 7 and (b) *L* = 13, *A* = 5, where *A*_*t*_ = *A* – 1. The estimated percolation thresholds are identified by dashed grey lines.

**Fig 5:**
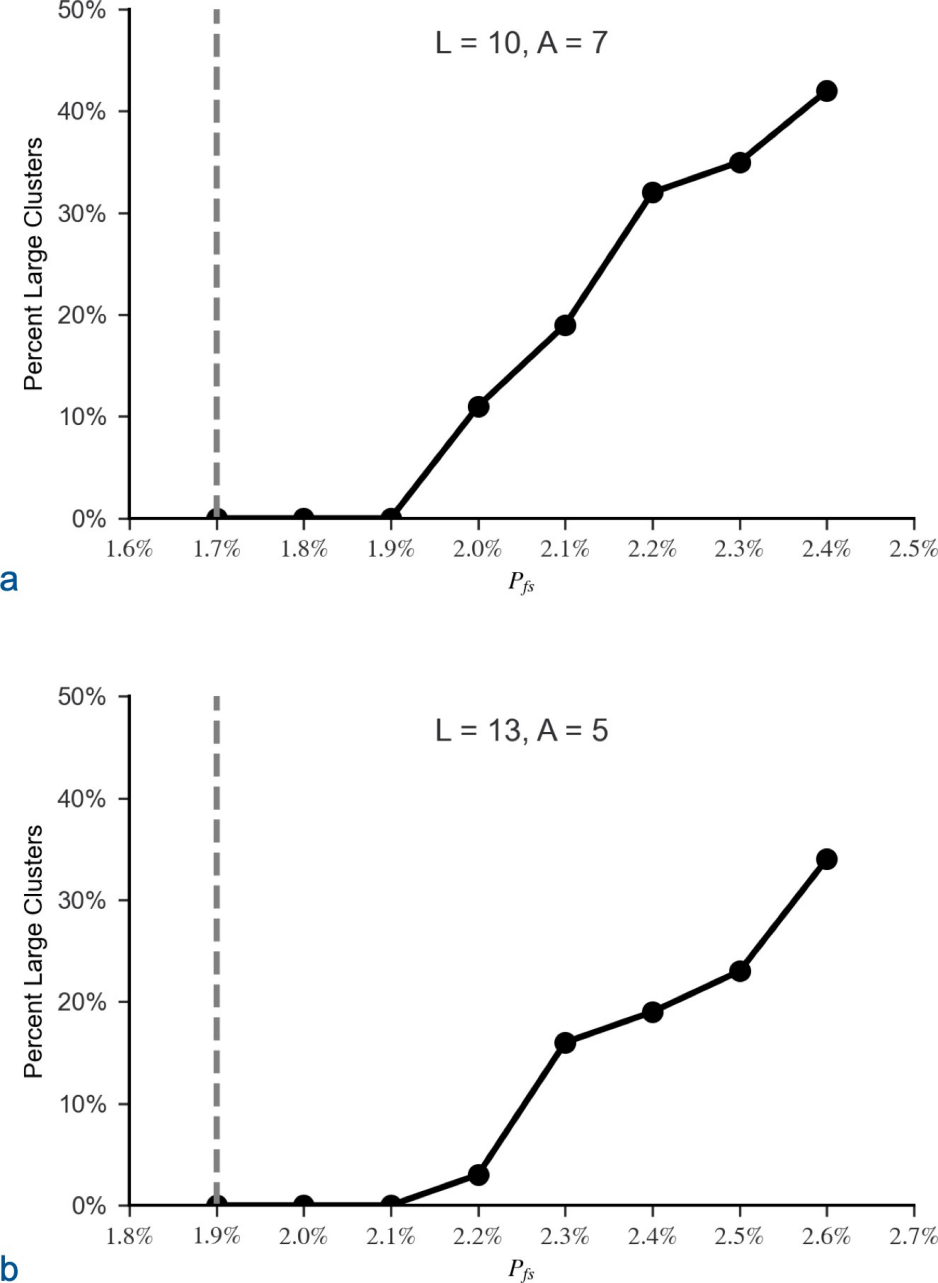
Percentage of starting sequences residing in large clusters. All clusters were either below 300 sequences or above 500,000 sequences. Due to the dramatic division between small and large clusters, the average percentage of starting sequences in large clusters was calculated for 100 matrices for each set of parameter values. No large clusters were identified until the proportion of function sequences, *P*_*fs*_, rose above a critical value roughly 0.2% above the estimated percolation threshold, *P*_*th*_ (Eq 2: *n*_*m*_ = 1), for both (a) *L* = 10, *A* = 7 and (b) *L* = 13, *A* = 5, where A_t_ = A – 1. The estimated percolation thresholds are identified by dashed grey lines.

**Fig 6:**
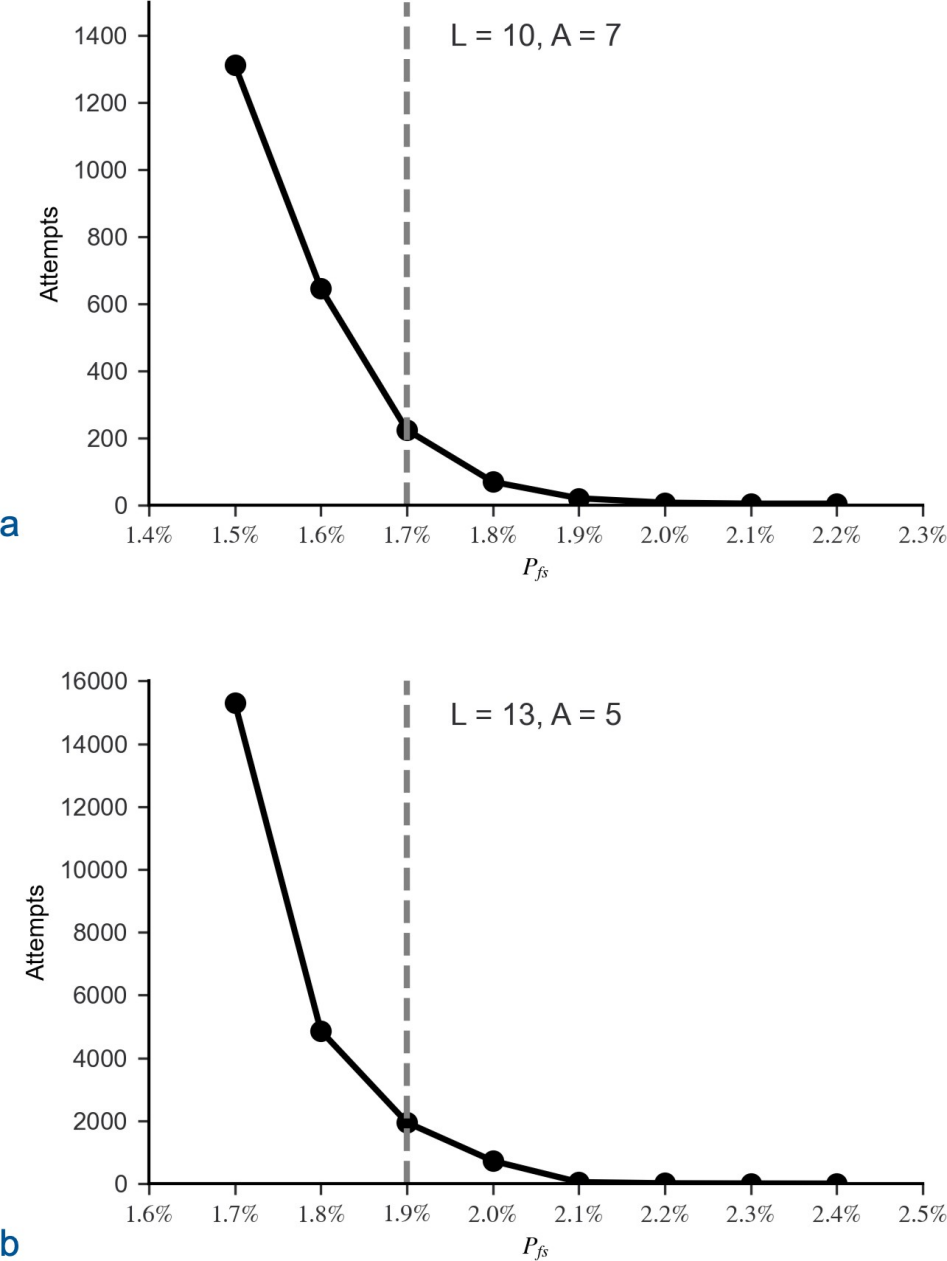
Average number of attempts required to generate a CFP between the starting sequence and the target. The target was all sequences that match a target sequence by all but at most 5 amino acids. Matrices were randomly generated until a continuous function path (CFP) connected the starting sequence to the target. The required number of attempts to generate a connecting CFP was averaged over 20 trials, where each attempt in a trial started with a matrix randomly populated with a specific proportion of functional sequences, *P*_*fs*_. The average number of required attempts grew quickly as *P*_*fs*_ decreased below a critical value, which is approximately 0.2% above the estimated percolation threshold, *P*_*th*_ (Eq 2: *n*_*m*_ = 1), for both (a) *L* = 10, *A* = 7 and (b) *L* = 13, *A* = 5, where *A*_*t*_ = *A –* 1. The probability of a sequence residing in a CFP that extends to the target approximates the reciprocal of the average number of attempts. The estimated percolation thresholds are identified by dashed grey lines.

The simulation was initially run with *L* = 10, *A* = 7 and *L* = 13, *A* = 5. The trials demonstrated the expected phase transition near *P*_*th*_ (Eq 2: *n*_*m*_ = 1). For *L* = 10, *P*_*th*_ = 1.7% and for *L* = 13, *P*_*th*_ = 1.9%. When *P*_*fs*_ dropped below a critical value approximately 0.2% above *P*_*th*_, clusters were small (Fig 4), and CFPs extending throughout sequence space ceased to exist (Fig 5). Correspondingly, the required number of attempts, *N*_*att*_, to generate a matrix with a CFP connecting the starting sequence to the target quickly increased when *P*_*fs*_ dropped below the same critical value approximately 0.2% above *P*_*th*_ (Fig 6).

All clusters comprised fewer than 300 sequences or greater than 500,000 sequences. A CFP that is part of the larger cluster class extends throughout sequence space, so it is designated an extensive continuous functional path. Identifying the presence of extensive CFPs is crucial since they are required for natural selection to assist the evolution of one protein into another distinct protein.

For the initial trials where *P*_*fs*_ = *P*_*th*_, the ratio of *N*_*att*_ to the size of the entire sequence space corresponded to roughly one successful attempt per million sequences in sequence space. This ratio is analogous to an ancestral protein’s *P*_*fs*_. The targets encompassed more than 0.1% of the sequence spaces, which is analogous to a descendent protein’s *P*_*fs*_. These proportions are far larger than the *P*_*fs*_ for even peptides that perform such simple functions as sticking to an ATP molecule. Moreover, the shortest path from the starting sequence to the target is only 5 steps for *L* = 10 and 7 steps for *L* = 13. Protein evolution and protein engineering entail searches in much larger spaces, involve much smaller *P*_*fs*_, and often require larger numbers of amino acid alterations. Consequently, the percolation thresholds observed in the simulation should not exceed the thresholds in actual protein sequence space.

Another set of trials was run to identify the critical value for the phase transition where large clusters appeared for the following parameters (*L*, *A*, *A*_*t*_): (7, 10, 9), (7, 20, 6), (7, 20, 19), (10, 5, 4), (10, 7, 2), (10, 8, 7). The transition for the largest sequence spaces was consistently close to 0.2% above the percolation threshold estimate (Eq 2). The transition was more than 0.2% above the estimate for trials with smaller *A* for a given *L*: (7, 10, 9) and (10, 5, 4). The transition also occurred at higher *P*_*fs*_ for trials that only allowed amino acids to transition to a limited set of possible amino acids: (7, 20, 6) and (10, 7, 2). The latter result was expected since allowing amino acids to transition to all other amino acids provides each sequence the easiest access to all of sequence space. A single mutation in proteins can result in changes to fewer than half of the available amino acids [23], further supporting that the simulation results do not underestimate the actual thresholds.

The results for all trials indicate that the phase transition occurs 0.2% or more above the percolation threshold estimate. The results should scale with *L* since they were consistent for *L* varying by almost a factor of two. This conclusion is further supported by theoretical analyses of complex multidimensional latices, which demonstrated that the percolation threshold never drops below the reciprocal of the number of nearest neighbors (Eq 1) but only approaches it as the number of nearest neighbors and the size of the lattice grows [24]. Consequently, *P*_*th*_ from Eq 2 represents a reliable lower bound to the percolation threshold for protein sequence space.

### Biasing and nonfunctional intermediates

Since *P*_*th*_ represents a conservative lower bound for the percolation threshold, *R*_*b*_ conservatively estimates the level of biasing required in the distribution of functional sequences in a region of sequence space for extensive CFPs to exist in that region. The *P*_*th*_ (*n*_*m*_ = 1), *P*_*fs*_, and *R*_*b*_ values for multiple peptides, polypeptides, and proteins are listed in Table 2. The data includes all commonly cited *P*_*fs*_ values. The *R*_*b*_ values for the proteins are exceedingly large, and they are significant even for the peptides and polypeptides.

The importance of the *R*_*b*_ values is clearer when they are connected to the lowest *n*_*m*_ required for *R*_*b*_ to drop below 1 (*P*_*th*_ to drop below *P*_*fs*_). The lowest *n*_*m*_ for the membrane embedding peptides is 4. Consequently, functional sequences are on average over 3 amino acid changes away from each other. The required *n*_*m*_ for the ATP binding polypeptides is 5 and much higher for the proteins. For a CFP to connect even functional peptides and polypeptides without significant biasing, individuals would have to tolerate multiple nonfunctional intermediate sequences before the next functional sequence was discovered along a CFP. Consequently, biasing must be extremely large for CFPs to connect different proteins (see Discussion).

Sources of sufficient biasing to allow for extensive CFPs have been identified. One source is proteins in nature (wildtype) being highly optimized for stability and function, so wildtype sequences are more tolerant to mutations than proteins that have already accumulated several amino acid changes [25–28]. The number of amino acid changes, *n*, a protein acquires is referred to as the Hamming distance from the wildtype sequence. *P*_*loc*_ is highest next to wildtype sequences and decreases with *n*, often faster than exponentially. For sufficiently small *n*, *P*_*loc*_ is larger than *P*_*th*_, allowing for extensive CFPs.

For some proteins, the decrease of *P*_*loc*_ with increasing *n* approximates a mathematical function, which can be designated *P*_*loc*_*(n)*. In such cases, *P*_*loc*_*(n)* can be set to *P*_*th*_ and solved for *n* to determine the maximum Hamming distance, *n*_*max*_, where *P*_*loc*_(*n*) still exceeds *P*_*th*_. The Hamming distance can be converted to the percentage of amino acids a sequence shares with the wildtype. This percentage is termed the sequence identity (SI), and the conversion follows a simple relationship:

$$SI=100\%-\frac{n}{L}.$$ (4)

These calculations were performed on the proteins β-lactamase, GFP, and HisA since their *P(n)* were reported or could be derived (see Methods). All *n*_*max*_ correspond to sequences that differ from the wildtype sequences by approximately 5% (Table 3), so sequences only have a significant probability of residing within an extensive CFP if their SI with a wildtype is around 95% or larger.

**Table 3:** Region neighboring wildtype sequences where *P*_*fs*_ is greater than *P*_*th*_. The percolation threshold, *P*_*th*_, was calculated from Eq 2 for *n*_*m*_ = 1 and *A*_*t*_ = 7.5. The maximum Hamming distances, *n*_*max*_, from wildtype sequences where *P*_*fs*_ > *P*_*th*_ were determined from experimental data. Data for β-lactamase comes from Bershtein et al. (2006), for GFP from Sarkisyan et al. (2016), and for HisA from Lundin et al. (2018). The maximum Hamming distances were converted to sequence identities (SI) using Eq 4. The region where *P*_*fs*_ > *P*_*th*_ for all the proteins is where their SI with a wildtype sequence is greater than approximately 95%.

Protein	Length	P_th_	n_max_	SI
β-lactamase	263	.00051	10–17	93.6–96.2%
GFP	236	.00056	12	95.0%
HisA	245	.00054	10	95.9%

## Methods

### Simulation

**Fig 7:**
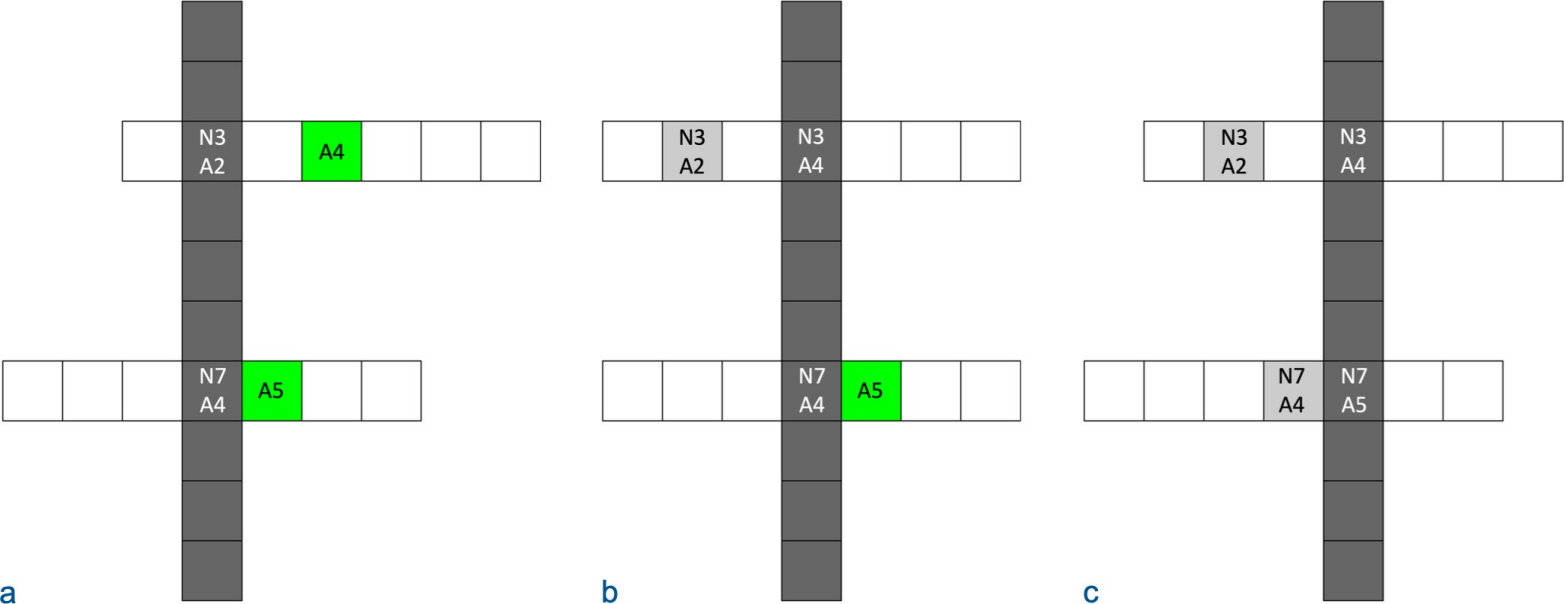
Sequence transitions in simulation. In this example, the sequence space corresponds to all sequences that are 10 amino acids long where each location in the sequence could hold one of seven possible amino acids. The positions in the sequence are labeled with *N*’s, and the number of the amino acid located at each position is labeled with *A*’s. The figure depicts two steps in a CFP. (a) The initial sequence has the 2^nd^ amino acid at the 3^rd^ position and the 4^th^ amino acid at the 7^th^ position. (b) The first mutation occurs at the 3^rd^ position, and it replaces the 2^nd^ amino acid with the 4^th^ amino acid. (c) The second mutation occurs at the 7^th^ position, and it replaces the 4^th^ amino acid with the 5^th^ amino acid. Each sequence is randomly assigned a value that determines if it is functional.

The simulation was programmed in Python and run on a Linux server using parallel computing. The search space is a matrix of dimension *L* where each dimension corresponds to a single location along the sequence. Every dimension has a size of *A*, which corresponds to the *A* possible amino acids that could reside at that location. For most trials, any amino acid could transition into any other amino acid, so *A*_*t*_ = *A*– 1 (Fig 7). Each cell in the matrix corresponds to a sequence, and it is assigned a random number between 0 and 1. If the value is below the *P*_*fs*_ designated for the sequence space, the sequence corresponding to the cell is classified as functional, else it is classified as nonfunctional. For instance, the cell [1, 3, 2] corresponds to the sequence (1^st^ amino acid, 3^rd^ amino acid, 2^nd^ amino acid). If *P*_*fs*_ = 5%, the cell would be functional if it were assigned a value below 0.05 and nonfunctional if assigned a value equal to or above 0.05.

A recursive algorithm started from the sequence consisting entirely of the 1^st^ amino acid and traversed every CFP for *n*_*m*_ = 1. For each matrix, the simulation either calculated the size of the cluster including the starting sequence or determined if a CFP connected the starting sequence and a target. The target corresponded to a target sequence consisting entirely of the 2^nd^ amino acid and the set of sequences that match it by all but at most a specified number of amino acids, designated *Tol* for tolerance.

Initial trials used the parameters *L* = 10, *A* = 7 or *L* = 13, *A* = 5 since they represent two of the largest sequence spaces the program could manage. *Tol* was set to 5 for both the *L* = 10 and *L* = 13 trials since the proportions of the sequence spaces contained in the targets were comparable. The code for the simulation and the output data are available on GitHub: https://github.com/drbjmiller/Sequence-Space.

For one set of trials, 20 matrices were generated in parallel with the same *P*_*fs*_, and the average size of clusters containing the starting sequence was recorded. Running 20 parallel processes was the maximum number the server could manage. Trials were run with *P*_*fs*_ values that ranged from 0.2% below *P*_*th*_ to 0.5% above *P*_*th*_. The simulation could only manage paths shorter than 15,000 sequences, so the cluster size output from the simulation became increasingly inaccurate after roughly 0.4% above *P*_*th*_.

For another set of trials, matrices were generated with the same *P*_*fs*_ until a CFP between the starting sequence and the target was identified. For each *P*_*fs*_, the average number of attempts was recorded. Trials were run with *P*_*fs*_ values that ranged from 0.2% below *P*_*th*_ to 0.5% above *P*_*th*_. Below the lower bound, the computational time required to generate a matrix with a connecting CFP grew extremely long.

Another set of trials was added due to the discovery that clusters were almost always either smaller than 300 sequences or larger than 500,000 sequences. The larger clusters extended throughout sequence space. Consequently, the percentage of starting sequences that are part of large clusters represents a more meaningful statistic than the average cluster size. For each *P*_*fs*_, 100 matrices were generated, and the percentage of large clusters was recorded. The exception was for *P*_*fs*_ = *P*_*th*,_ where 100,000 matrices were generated with a modified version of the simulation that stopped searching a matrix when the cluster size exceeded 20,000 sequences, since all clusters over that size extended throughout sequence space. Trials were run with *P*_*fs*_ values that ranged from 0.2% below *P*_*th*_ to 0.5% above *P*_*th*_. No large clusters were observed below *P*_*th*_.

The graphs of the percentage of large clusters clearly identified the percolation phase transitions for initial trials, so the modified version of the simulation was used to identify the phase transition for additional trials to ensure that the results did not vary significantly with *L* or *A*. The modified simulation was then further modified to only allow amino acids to transition to a limited number of other amino acids to ensure results did not vary significantly with *A*_*t*_. The results from all trials are accessible in the GitHub repository.

### Comparing *P*_*th*_ to *P*_*fs*_

The commonly cited *P*_*fs*_ values for peptides, polypeptides, and proteins were used in this study. Knopp et al. (2019) provided the data for membrane embedding proteins, Keefe et al. (2019) for ATP binding polypeptides, Taylor et al. (2001) for chorismite mutase, Reidharr-Olson and Sauer (1990) for λ-repressor, Yockey (1977) for cytochrome c, Axe (2004) for the larger domain of TEM-1 β-lactamase, and Tian and Best (2017) for the other 10 single-domain proteins. The source for each *P*_*fs*_ value is cited in Table 2. *P*_*th*_ was calculated for each entry using Eq 2 with *n*_*m*_ = 1 and *A*_*t*_ = 7.5 since that value is the average number of possible amino acid transitions resulting from a single mutation [23].

### Determining *P*_*loc*_*(n)*

The *P*_*loc*_*(n)* functions were derived from the empirical data for the proteins TEM-1 β-lactamase from *E*. *coli* [25], GFP from *Aequorea victoria* [26], and HisA from *Salmonella enterica* [27]. The maximum Hamming distance, *n*_*max*_, from a wildtype sequence where *P*_*loc*_*(n)* > *P*_*th*_ was determined for each protein. The value of *n*_*max*_ for β-lactamase was initially derived from Table 1 in Bershtein et al. (2006), which lists the percentage of tolerated nonsynonymous mutations after increasing rounds of mutagenesis with selection between rounds. The value for *P*_*loc*_*(n)* was estimated by multiplying the highest percentage of tolerated mutations, *P*_*tol*_, in the listed range for each added mutation. Specifically, *P*_*tol*_ was set to 100% for the first mutation, since *P*_*tol*_ was not listed, 61% for the next two mutations, 55% for the next two, and 39% for subsequent mutations yielding *n*_*max*_ = 10.

The investigators also measured the percentage of functional proteins with different numbers of mutations for trials not applying selection between rounds. They determined *P*_*loc*_*(n)* by fitting the data to different functions. It best fit a decaying hyper-exponential:

$$P_{loc}(n)=e^{-\alpha n-\beta n^{2}}.$$ (5)

The investigators included all mutations in their analysis, so I rescaled the variable *n* to only include mutations that altered the sequence (nonsynonymous) by replacing *n* with *n*/0.69 yielding α = 0.104 and β = 0.019. Setting the equation equal to β-lactamase’s *P*_*th*_ yields *n*_*max*_ = 17. The actual *n*_*max*_ is likely somewhere between the two estimates. The lower value is likely preferable since the context is environments undergoing high levels of selection.

*P*_*loc*_*(n)* for GFP was derived by curve fitting the reported data on *P*_*loc*_*(n)* for *n* between 2 and 10 to Eq 5 using the function *optimize*.*curve_fit* from the *SciPy* Python library. The data best fit with α = -0.062 and β = 0.058. The hyper-exponential function is the standard choice for proteins that display pervasive negative epistasis [29], which the investigators reported. The data fit the equation very well except for *n* = 1. This value represents such an extreme outlier that it was not included in the curve fitting. Setting *P*_*loc*_*(n)* to GFP’s *P*_*th*_ yields *n*_*max*_ = 12.

The value of *n*_*max*_ for HisA was derived from the “selection” function reported by Lundin et al. (2018), which is also a hyper-exponential function with α = 0.165 and β = 0.065. The selection function represents the relative growth rate of bacteria with mutated HisA proteins. The function is not identical to *P*_*loc*_*(n)*, but the rapid loss of function with accumulating mutations [28] suggests that the selection function serves as a reasonable proxy since both functions approach 0 at the same time. HisA’s *P*_*loc*_*(n)* approaches its *P*_*th*_ around *n*_*max*_ = 10.

## Discussion

### Biasing of distribution of functional sequences

Since many proteins correspond to functional sequences that are too rare for them to have been discovered through a random search, they could only have originated from ancestral protein sequences connected by CFPs to modern protein sequences [30]. A modern protein could either be a protein found in nature today or a newly engineered protein. The ancestral protein would then be either the earlier protein that evolved into the modern protein or the initial protein and was engineered into the new protein respectively. This study quantified the level of biasing required in the distribution of functional sequences in sequence space for extensive CFPs to exist.

If artificial or natural selection is constraining the evolution of a protein, the average distance between two functional sequences often could not exceed one mutation since nonfunctional intermediates would be quickly removed from the population under sufficiently high selection to constrain an evolutionary path along a CFP. Conversely, the time required for two specific mutations to arise in an individual when possessing only one is disadvantageous is often prohibitively long [31].

A *n*_*m*_ = 2 CFP could realistically constrain an evolutionary search for species with very large populations since two coordinated mutations would occur in individuals sufficiently often even if possessing only one were detrimental. For instance, *P*. *falciparum* acquires two coordinated mutations that impart resistance to chloroquine with a per-parasite probability on the order of 1 in 10^20^ parasite multiplications [32]. The number of multiplications per year for many species is larger than 10^20^.

Only in species with the largest populations over geological timescales could *n*_*m*_ = 3 CFPs constrain an evolutionary search. Natural selection would much more often remove mutations that disabled a protein before two additional mutations reactivated it, and the number of multiplications required to obtain three simultaneous specific mutations is on the order of 10^30^ based on the number required for two. Even without purifying selection, the number of coordinated mutations that could spread through a population is less than 10 under any circumstance [33]. The value of *P*_*th*_ does not drop below *P*_*fs*_ for any protein referenced in this study even for *n*_*m*_ = 10 (Table 2).

One might postulate that indels could substantially lower *P*_*th*_ since they greatly increase the number of potential neighboring sequences. Yet the low ratio of documented indels to single nucleotide changes in the coding regions of DNA in diverse taxa [34] indicates that indels should not significantly decrease the effective *P*_*th*_. They certainly would not decrease *P*_*th*_ sufficiently to drop below the cited protein *P*_*fs*_ values.

Proteins’ consistently high *R*_*b*_ even for unrealistically high *n*_*m*_ indicate that the distribution of functional sequences must typically be extremely biased for CFPs to assist evolutionary searches. Such strong biasing occurs in regions of sequence space close to wildtype sequences. Mutation studies of multiple proteins reported *P*_*loc*_ remaining above the percolation threshold in regions where sequences do not differ from a wildtype by more than approximately 5%. At farther Hamming distances, the biasing must result from proteins possessing special properties that result in narrow tendrils extending through sequence space where *P*_*loc*_ > *P*_*th*_.

This prediction supports the conclusion of previous studies that proteins capable of evolving new functions naturally or through engineering have highly optimized structures to generate such biasing. In many cases, different CFPs branching from a protein’s wildtype sequence have been shown to lead to the protein performing different functions at high efficiency without significantly altering its overall structure [35, 36]. In what are termed promiscuous enzymes, mutations can modify the active site to enable or enhance many possible catalytic activities, allowing an organism to adapt to new environments or a protein engineer to achieve multiple target functions [37]. For instance, a β-lactamase enzyme in *Arthrobacter sp*. gained the ability to digest human-manufactured nylon by acquiring only two mutations [38].

The consistently high *R*_*b*_ values calculated in this study further support the conclusion that proteins’ ability to evolve new functions results from highly specialized structural features. In addition, a protein’s *R*_*b*_ helps quantify the level of specification required in its sequence and structure to enable its evolvability.

### Improvements to methodologies

As mentioned, Buchholz et al. (2017) applied percolation theory to protein sequence space, but they failed to properly incorporate the percolation threshold. They analyzed the distribution of the size of clusters of functional protein sequences in six superfamilies [39]. For each superfamily, they identified a protein sequence as a member of a cluster if it neighbors another sequence in the cluster based on the criterion that its SI with the neighbor is above a set cutoff. The investigators observed that the number, *N*, of clusters of size *s* follows a power-law with exponent -τ:

$$N(s)∼s^{-\tau }.$$ (6)

They argued that this observation is a direct expectation of percolation theory.

For each superfamily, they calculated τ for cutoffs that ranged from 60% SI to 90% SI. They found that τ increases linearly with increasing SI. They then extrapolated the linear relationship to estimate τ for clusters where neighbors were separated by a single amino acid (i.e., SI approaching 100%), so clusters represent interlinking extensive CFPs. Based on this result, the investigators concluded that CFPs should interconnect all proteins in a superfamily since *N(s)* remains finite for large *s*.

Yet this extrapolation is not justifiable. The authors assumed that the power-law behavior would apply for all SI, but it only applies when *P*_*fs*_ is not significantly below *P*_*th*_. Below the percolation threshold, extensive CFPs cease to exist. For 60% sequence identify, *n*_*m*_ is often sufficiently large that *P*_*fs*_ is greater than *P*_*th*_ (Eq 2), but for cutoffs approaching 100% *P*_*th*_ greatly exceeds *P*_*fs*_. The SI cutoff for the proteins included in this study are listed in Table 2. The frequency of extensive CFPs for *P*_*fs*_ below the percolation threshold cannot be determined by extrapolating results from data corresponding to *P*_*fs*_ above the threshold.

A better approach for studies of protein sequence space is to first determine *P*_*th*_, *P*_*fs*_, and *P*_*loc*_*(n)* for proteins under investigation and then incorporate the percolation phase change into analyses by treating the different regimes independently. In the region below the threshold, investigators could use *R*_*b*_ to determine the level of required biasing of the distribution of functional sequences to allow for extensive CFPs. They could also identify where the biasing results from the proximity of sequences to wildtype proteins (i.e., small Hamming distances) and where it results from proteins being optimized for easily developing new catalytic functions.

The source of the biasing could be explored by such tools as the sequence evolution with epistatic contributions (SEEC) model developed by Alvarez et al. (2021), which identifies evolutionary relevant epistatic interactions between amino acids [40]. The model’s developers used it to guide the modification of enzymes to effectively explore sequence space to enhance target functions. A similar tool was developed by Durston et al. (2012) that employs a *k*-modes attribute clustering algorithm to connect sets of amino acids to a protein’s structure and function [41]. Such tools could help identify how a protein’s adaptability–and by extension the biasing of sequence space–is facilitated by sequence and structural motifs.
